# Case of Strychnine Poisoning

**Published:** 1874-01

**Authors:** 


### Case of Strychnine Poisoning.

On September 19th an inquest was held by Dr. R. F. Dill, the coroner for Belfast, on the body of Thomas Shaw, aged thirty-seven, who had met his death three days previously from taking an overdose of strychnine. From the somewhat imperfect report of the inquest, and from the newspaper correspondence to which it has given rise, we gather the following facts: The deceased, who was foreman in a muslin factory, was a delicate man and a great smoker, and possibly also (but of this there is doubt) was the subject of heart disease. On the 15th of September he consulted a medical man for some slight derangement of vision, who prescribed for him "seven drops of liquor strychniæ three times daily in water after meals. This prescription was dispensed by Messrs. Grattan & Co., who, it appears, in accordance with their instructions, furnished the deceased with an ounce bottle filled with liquor strychniæ of Pharmacopœial strength. From this bottle the patient was directed to measure his own dose of seven drops by dropping into a wineglass. On September 16th, after breakfast, the deceased took a dose of his medicine without any ill effects. On the same day, after dinner, about twenty minutes after two, he took a second dose, and his wife, who saw him take it, counted the seven drops "in a run on the glass." He then took a short turn in the yard, and came in again "to take a nap on the sofa." He had not lain five minutes when he gave a yell, and jumped up, saying that he was poisoned, and asked for hot water, of which he drank three tumblersful. He vomited and became convulsed, the convulsions being tetanic in character, so that they were at once recognized as such by the medical man who was called in; the pupils were largely dilated; and he died in convulsions within an hour of his taking the fatal dose. The examination of the bottle from which the dose had been taken showed that more than 120 minims were wanting to make the total quantity which the bottle was originally supposed to contain.

Supposing the patient to have taken only fourteen minims of the solution, the dose of strychnine amounted to rather more than one-eight of a grain in five or six hours, a dose far short of that which hitherto has been recognized as a fatal one, for half a grain is spoken of as the smallest fatal dose for an adult. If the patient took 120 minims, then he received a grain of strychnine in the specified time, and it is highly probable that the last dose was more than ample to destroy life. There are, however, several links missing in the chain of evidence. First, it was not proved that the bottle of solution as furnished to the deceased was absolutely full, so that the quantity missing from the bottle after the accident is hardly a safe criterion of the dose of strychnine taken. Secondly, it was not proved by analysis that the solution with which the patient was provided was actually of the Pharmacopœial strength. The lesson to be learnt from this case is the unadvisability of prescribing potent poisons in so concentrated a form. Here we have the history of a patient being furnished with twenty-two days' medicine—sufficient, possibly, to kill eight men—in so small a compass that he might carry it in his waistcoat-pocket. Then, again, the mode of measuring the dose is one which gives most varying results. We have seen sixty drops of water let fall from a bottle into a minim measure fill the said measure to the amount of two drachms and a half; so that it seems to us possible that, even supposing the deceased counted his dose accurately, he may have received more than twice the amount which was intended for him. We heard not long since of a similar (though not fatal) accident occuring to a London physician who had prescribed a mixture containing strychnine, with the direction that a teaspoonful was to be taken for a dose. On revisiting his patient, he was horrified at finding him suffering from symptoms of strychnine poisoning, which had been brought about by the not unnatural mistake of the druggist, the nurse, or the patient—we forget which—of a "tablespoon" for a "teaspoon." Dangerous drugs like strychnine should always be administered in the most bulky form which is compatible with the well-being of the patient.—*London Lancet.*

